# The effect of steroids used in the treatment of coronavirus disease 2019 on infections in intensive care

**DOI:** 10.14744/nci.2022.43827

**Published:** 2022-04-13

**Authors:** Ahmet Sari, Mesut Aslan, Osman Ekinci

**Affiliations:** Department of Anesthesiology and Reanimation ICU, University of Health Sciences, Haydarpasa Numune Training and Research Hospital, Istanbul, Turkey

**Keywords:** COVID-19, cytokine storm, intensive care, secondary infections, steroids

## Abstract

**Objective:**

Cytokine storm in coronavirus disease 2019 (COVID-19) patients causes lung damage and acute respiratory distress syndrome (ARDS). Immunomodulators such as steroids are widely used to control this situation. This study investigates the effectiveness of steroids used in COVID-19 patients, and their effects on secondary infections, morbidity, and mortality.

**Methods:**

Data were obtained by retrospectively scanning the files of patients in our hospital’s intensive care unit clinic during the three peak periods.

**Results:**

Between the steroid and non-steroid groups, there was no statistically significant difference in reproductive rates. These rates were 49.7% and 43.2%, respectively. Reproductive rates among steroid types were determined as 25 (56.8%) in the Methylprednisolone group, 18 (69.2%) (Highest) in the Dexamethasone + Methylprednisolone group, and 54 (43.2%) (Lowest) in the Dexamethasone group. Steroid treatment duration was effective on reproduction. Steroids cause more infections, especially after invasive procedures (Tracheal intubation, central venous catheter, etc.). In the groups with and without tracheal aspirate steroids, the growth rates were 71 (76.3%) and 32 (54.2%) respectively. There was no difference in mortality between the groups.

**Conclusion:**

Cytokine storm causes lung damage and ARDS. Steroids can be useful in controlling this hyper-inflammatory situation. However, increased secondary infections, an important side effect of steroids, increase mortality. Steroids more often cause these infections, especially in patients undergoing invasive Strict adherence to infection control measures during steroid treatment will reduce this risk. In conclusion, while steroids reduce mortality by controlling the hyper-inflammatory picture, they also increase mortality with increased secondary infections. Preventing infections enables success with steroids.

**C**oined as the “novel coronavirus,” coronavirus disease 2019 (COVID-19) is a disease caused by the severe acute respiratory syndrome coronavirus 2 (SARS-CoV-2). After COVID-19 first emerged in Wuhan, China in late 2019, it was declared a pandemic by the World Health Organization (WHO) on March 12, 2020. As of May 15, 2021, approximately 162 million people were infected and unfortunately, 3.35 million people were lost to the disease [1]. Irregular host response to lung infection, which develops in association with SARS-CoV-2, together with immune-mediated and pro-inflammatory cytokine storm, lead to lung damage and the emergence of acute respiratory distress syndrome (ARDS) [2, 3]. Cytokine storm causes excessive and/or uncontrolled release of pro-inflammatory cytokines, it advances as a result of the complex and successive activation of many cells, resulting in ARDS and multiple organ failure [4]. Fighting against this new virus, different treatments such as favipiravir, hydroxychloroquine, azithromycin, Vitamin C, immune plasma, and tocilizumab were administered in the first peak period. While some of these treatments are still administered, some are no longer used. Researches found that hyper-inflammation played an important role in the clinic aftermath, hence subsequent researches focused on taking control of hyper-inflammation using immunomodulators such as corticosteroids as a treatment option for COVID-19. According to studies conducted for this purpose, steroids are effective in COVID-19 patients and reduce mortality [2, 5, 6]. There are also studies [7, 8] reporting that steroids’ effect is limited, but steroids have been used more especially in the second peak. Steroids have positive effects on cytokine storm, as well as negative effects. Especially the increase in the tendency towards secondary infections may lead to increased morbidity and mortality.

COVID-19 patients admitted to intensive care suffer from advanced respiratory failure, and these patients need more invasive monitorization and respiratory support. Each invasive procedure administered poses an infection risk. The dysregulated immune response that develops in the host, invasive procedures, and the immunosuppressive agents we administer in treatment increase this risk.

## Materials and Methods

After the start of the COVID-19 epidemic, our treatment strategies in the first and second peaks were shaped according to the developments in the process. Steroids, which we almost never used in the second peak, started to be used more in intensive care units (ICU) after their positive effects were demonstrated in the second peak. However, steroids can also exhibit negative effects. The most important of these is secondary infections that may develop. Our aim in this study is to investigate the efficacy of steroids, which we administer more frequently to our patients in the second and third peaks, and their effects on secondary infections, after obtaining institutional and Haydarpasa Numune Training and Research Hospital Clinical Research Ethics Committee permissions (HNEAH-KAEK 2021/12). For this purpose, the files of our patients hospitalized in both peaks were scanned retrospectively, their blood, endotracheal aspirate (ETA), and catheter cultures were studied and the results were recorded. Reproduction instances compatible with clinic and/or laboratory were regarded as cause of infection. Patients who were administered steroid treatment in the ICU were included in the study. For our patients, steroid treatment was administered in the form of dexamethasone 6mg/day and/or mini pulse methylprednisolone (250 mg/3 days and 80 mg/7 days as maintenance) according to clinic.

**Highlight key points**

• Reproductive rates among steroid types were determined as 25 (56.8%) in the Methylprednisolone group, 18 (69.2%) (highest) in the Dexamethasone+Methylprednisolone group and 54 (43.2%) (lowest) in the Dexamethasone group.
• Steroid treatment duration was effective on reproduction
• Steroids cause more infections, especially after invasive procedures (tracheal intubation, central venous catheter, etc.). In the groups with and without tracheal aspirate steroids, the growth rates were 71 (76.3%) and 32 (54.2%) respectively.
• There was no difference in mortality between the groups.

### Statistical Reviews

When evaluating the findings obtained in the study, IBM SPSS Statistics 22 (SPSS IBM, Turkey) programs were used for statistical analyses. While evaluating the study data, the appropriateness of the parameters to normal distribution was evaluated using the Shapiro Wilks test. Descriptive statistical methods (Mean, Standard deviation, frequency) were used while evaluating the study data. In addition to these, Student t test was used for comparing quantitative data between the two groups with normally distributed parameters. Mann Whitney U test was used for comparisons between two groups that did not exhibit normal distribution. Chi-square test, Fisher’s Exact test, and Continuity (Yates) Correction were used for comparison of qualitative data. Significance was evaluated at p<0.05 level.

## Results

Our study was conducted between March 2020 and January 2021 with a total of 306 subjects, of which 184 (60.1%) male and 122 (39.9%) female, aged between 19 and 99. The average age of the subjects is 67.0±16.6. The study was examined under two groups, namely 195 (63.7%) steroid and 111 (36.3%) non-steroid patients (Table 1).

**Table 1.** Evaluation of age, gender, steroid type, mechanical ventilation, antibiotics, steroid, sedation, and MV duration between the groups

	Steroid (Min-Max)-(Mean±SD)	Non-Steroid (Min–Max)-(Mean±SD)	p
Age	(19–93)–(64.51±16.40)	(21–99)–(71.41±16.22)	^1^0.000*
Antibiotic–treatment time (days) (median)	(0–29)–(8.23±6.13 (8))	(0–27)–(6.53±4.55 (6))	^2^0.021*
Steroid treatment duration (days) (median)	(1–23)–(8.35±4.04 (9))	–	–
Sedation duration (days) (median)	(0–30)–(5.56±6.75 (3))	(0–28)–(4.23±5.97 (2))	^2^0.254
MV duration (median)	(0–38)–(6.74±8.06 (4))	(0–35)–(5.67±7.24 (4))	^2^0.777
		n=195 (%)	n=111 (%)	
Gender			
Male	63.6	54.1	^3^0.101
Female	36.4	45.9	
Steroid type			
Dexamethasone	64.1	–	–
Methylprednisolone	22.6	–	
Dexamethasone+methylprednisolone	13.3	–	
Mechanical ventilation			
Not applied	33.8	21.6	^3^0.024*
Applied	66.2	78.4	
ICU discharge			
Ex	56.4	67.6	^3^0.055
Alive	43.6	32.4

1: Student t-test; 2: Mann Whitney U Test; 3: Chi-square test; *: P<0.05; ICU: Intensive care unit; MV: Mechanical ventilation; SD: Standard deviation; Min: Minimum; Max: Maximum.

The mean ages of the steroid group were statistically significantly lower than those of the non-steroid group, and antibiotic durations were found to be statistically significantly higher (p=0.021; p<0.05).

The rate of mechanical ventilation (MV) administration in the Non-Steroid group (78.4%) was statistically significantly higher than that in the Steroid group (66.2%) (0:0.024; p<0.05) (Table 1). There is no statistically significant difference in terms of mortality rates between steroid and non-steroid groups (p>0.05) (Table 1).

There was no statistically significant difference between mortality rates according to steroid types (p>0.05) (Table 1). When we compared the two groups in terms of comorbidities, the comorbidity rate (77.3%) in the Steroid group was found to be statistically significantly lower than that in the Non-Steroid group (89.2%) (p=0.015; p<0.05). Dementia, malignancy, and atrial fibrillation rates were found to be lower in the steroid group than in the non-steroid group. There was no statistically significant difference between the groups in terms of the incidence of chronic obstructive pulmonary disease, congestive heart failure, coronary artery disease, diabetes mellitus (DM), chronic kidney failure, central venous diseases, hyperlipidemia, hypothyroidism, and benign prostatic hyperplasia (p>0.05).

The rate of reproduction in the steroid group was 49.7%, and 43.2% in the Non-Steroid group and there was no statistically significant difference between the groups (p>0.05) (Table 2). There was no difference in reproduction rates in the samples sent from blood and urine in both groups. The reproduction rates in the samples sent from ETA were (76.3%) and (54.2%) in the steroid and non-steroid groups, respectively, and the reproduction rates in the samples sent from the catheter were still were found to be significantly higher in the steroid group, respectively (73.7%) and (20%) (Table 2).

**Table 2.** Evaluation of reproductive, blood, urine, ETA and catheter sampling and reproduction instances between groups

	Steroid		Non-Steroid		p
	n	%	n	%	
Reproduction presence					^1^0.274
	No	98	50.3	63	56.8
	Yes	97	49.7	48	43.2	
Blood sampling status					–
	Not taken	68	34.9	48	43.2
	Taken	127	65.1	63	56.8	
Reproduction status in blood (n=190)					^1^0.865
Not observed	77	60.6	39	61.9
Observed	50	39.4	24	38.1	
Urine sampling status					–
	Not taken	138	70.8	94	84.7
	Taken	57	29.2	17	15.3
Reproduction status in urine (n=74)					^2^1.000
Not observed	39	68.4	11	64.7
Observed	18	31.6	6	35.3
Sampling from ETA					–
Not taken	102	52.3	52	46.8
Taken	93	47.7	59	53.2
Reproduction status in ETA (n=152)					^1^0.004*
Not observed	22	23.7	27	45.8
Observed	71	76.3	32	54.2
Sampling from catheter					–
Not taken	176	90.3	106	95.5
Taken	19	9.7	5	4.5
Reproduction status in catheter (n=24)					^3^0.047*
Not observed	5	26.3	4	80
Observed	14	73.7	1	20

1: Chi-square test; 2: Continuity (Yates) correction; 3: Fisher’s exact test; *: P<0.05; ETA: Endotracheal aspirate.

The duration of steroid treatment in individuals, where reproduction was observed, was found to be statistically significantly longer than those with no reproduction (p=0.040; p<0.05) (Table 3).

**Table 3.** Evaluation of the relationship between the presence of reproduction, and steroid treatment duration and steroid types

	Reproductive		p
	No	Yes	
Steroid treatment duration (days) Mean±SD (median)	7.65±3.54 (8)	9.06±4.39 (9)	^1^0.040*
Steroid type, n (%)			
Dexamethasone	71 (56.8)	54 (43.2)	^2^0.031*
Methylprednisolone	19 (43.2)	25 (56.8)	
Dexamethasone+methylprednisolone	8 (30.8)		18 (69.2)

1: Mann Whitney U test; 2: Chi-square test; *: P<0.05; SD: Standard deviation.

A statistically significant difference was found between steroid types in terms of reproduction rates (p>0.031; p<0.05). In the Dexamethasone + Methylprednisolone group reproductive rate (69.2%) was significantly higher than in the Dexamethasone group (43.2%) (p=0.028; p<0.05). There was no significant difference between other steroid types (p>0.05) (Table 3). The rate of growth in Ex patients was 75 (68.2%) and 38 (50.7%) in the steroid and non-steroid groups, respectively. In surviving patients, these rates were 22 (25.9%) and 10 (27.8%), respectively, hence statistically significantly higher (p=0.000; p<0.05) (Table 4).

**Table 4.** Evaluation of reproductive status in groups according to the type of respective ICU discharge status

Group	Repr.	ICU discharge				p
		Ex		Alive		
		n	%	n	%	
Steroid	No	35	31.8	63	74.1	0.000*
	Yes	75	68.2	22	25.9
Non-Steroid	No	37	49.3	26	72.2	0.038*
	Yes	38	50.7	10	27.8

Continuity (Yates) Correction; *: P<0.05; ICU: Intensive care unit.

Compared in terms of reproductive factors, no statistically significant difference was found among the groups in terms of *Acinetrobacter*, *Staphylococcus aureus*, *Klebsiella*, *Pseodomonas*, *Escherichia coli*, *Enterobacter*, *Staphylococcus epidermidis*, *Sterotrop-homonas* and *Staphylococcus hominis* reproduction (p>0.05) (Table 4).

In patients undergoing MV, the mortality rate in the steroid and non-steroid groups were (84.5%) and (86.2%), respectively. In patients who did not undergo MV, the same rates were (1.5%) and (0%), respectively, and hence statistically significantly higher (p=0.000; p<0.05) (Table 5). Among the patients who underwent MV, the mortality rate (63.3%) in patients with comorbidity in the steroid group was found to be statistically significantly higher than those who did not (31.8%) (p=0.000; p<0.05). In the non-steroid group, there was no statistically significant difference in mortality rates between those with and without comorbidity (p>0.05) (Table 5).

**Table 5.** Evaluation of ICU discharge type according to mechanical ventilation and comorbidities in groups separately

Group	ICU discharge		p
	Ex n=110 (%)	Alive n=85 (%)	
Steroid			
Mechanical ventilation			^1^0.000*
Not applied	1.5	98.5	
Applied	84.5	15.5	
Comorbidity			^1^0.000*
No	15	68.2	
Yes	95	36.7	
CHF			^1^0.710
No	57.1	42.9	
Yes	50	50	
COPD			^1^0.142
No	54.1	45.9	
Yes	72	28	
HT			^3^0.040*
No	49.5	50.5	
Yes	64.1	35.9	
DM			^1^0.006*
No	49.6	50.4	
Yes	72.4	27.6	
CRY			^1^0.673
No	55.6	44.4	
Yes	62.5	37.5	
Malignancy			^1^0.546
No	55.3	44.7	
Yes	64	36	
Non-Steroid			
Mechanical ventilation			^1^0.000*
Not applied	0	100	
Applied	86.2	13.8	
Comorbidity			^2^1.000
No	66.7	33.3	
Yes	67.7	32.3	
CHF			^1^0.722
No	66.3	33.7	
Yes	73.7	26.3	
COPD			^2^0.770
No	66.7	33.3	
Yes	73.3	26.7	
HT			^1^0.818
No	69.1	30.9	
Yes	65.1	34.9	
DM			^1^0.259
No	71.1	28.9	
Yes	57.1	42.9	
CRY			^1^0.722
No	66.3	33.7	
Yes	73.7	26.3	
Malignancy			^1^0.435
No	65.1	34.9	
Yes	76	24

1: Continuity (Yates) Correction; 2: Fisher’s Exact Test; 3: Chi-square test; *: P<0.05; COPD: Chronic obstructive pulmonary disease; CHF: Congestive heart failure; HT: Hypertension; DM: Diabetes mellitus; ICU: Intensive care unit.

In patients undergoing MV, the mortality rate in the steroid and non-steroid groups were (64.1%) and (72.4%), respectively. In patients who did not undergo MV, the same rates were (49.5%) and (49,6%), respectively, and hence statistically significantly higher (p=0.040; p<0.05). In the non-steroid group, there was no difference for either disease (Table 5). There was no difference between the two groups in terms of other comorbidities (Table 5).

## Discussion

Patients admitted to intensive care due to COVID-19 suffer from advanced respiratory failure. Respiratory support is provided to these patients in the ICU using different means such as high flow nasal oxygen, noninvasive MV, and invasive MV (IMV). One of the most important points in the deterioration of the clinic status and the course of ARDS in COVID-19 patients is the cytokine storm triggered by COVID-19 infection. One of the agents that can help control this situation is steroids. However, steroids can also exhibit negative effects. The most important of these side effects are secondary infections which cause significant increases in morbidity and mortality. Highly heterogeneous results were reported in regards to steroid use on COVID-19 patients. Although some studies suggest that steroids increase secondary infections and mortality [9, 10], there are also studies which report that steroids do not increase the rate of secondary infection, but they can be ineffective [11] or effective [12] likewise. In our study, reproductive rates were slightly higher in the group that received steroids, 49.7% and 43.2%, respectively, compared to the group that did not. However, there was no difference in terms of mortality rates. In terms of diabetes, the infection rates in patients without diabetes were found to be at the same level at 45.9% and 45.7% in the steroid and non-steroid groups, respectively. Infection rates in diabetic patients were determined as 58.6% and 32% in steroid and non-steroid groups, respectively. We believe that steroids administered in patients with diabetes should be used more carefully, as they may cause more infections. The Yang et al. [13] and Zhang et al. [14] studies found the infection rates after tracheal intubation to be 58.3% and 30.43%, respectively. In our study, we found that this rate was 54.2% in the non-steroid group, and 76.3% in the steroid group, which was much higher. The Zhang et al. [14] study reports that invasive MV support and intravascular devices increase the risks for secondary infections and therefore may be associated with high mortality. In our study, we found that invasive procedures (tracheal intubation, central venous catheter, dialysis catheter, etc.) increased secondary infections in both groups. We can say that steroids cause more infections, especially in patients who have undergone invasive interventions, since there is reproduction in 76.3% of tracheal aspirates sent from those intubated in steroids, and in 73.7% of blood cultures sent from the catheter. We believe that more strict adherence to infection control measures when steroids are used in these patients will contribute to the reduction of these rates and decrease mortality.

It was suggested that in terms of the efficacy of steroid types used in the treatment of COVID-19 patients, hydrocortisone is not effective [11], dexamethasone is effective [12, 15] and methylprednisolone is more effective, in a study which compared methylprednisolone and dexamethasone [16]. In our study, no statistically significant difference was found in terms of the effects of steroid types on mortality, but mortality occurred in 65 subjects (52%) in the group where dexamethasone was used, compared to 28 subjects (63.6%), a lower value, in the group where methylprednisolone was used. In our study, no difference was found in terms of mortality between the steroid group and the non-steroid group. The van Paassen et al. [17] and Ranzani et al. [18] studies reported that steroid use increases the tendency to secondary infections. In our study, secondary infections were observed at a higher level of 49.7% in the steroid group. We found that the duration of steroid administration is effective in the development of secondary infections. In addition, the effects of Dexamethasone+Methylprednisolone, methylprednisolone, and dexamethasone on reproductive rates were 69.2%, 56.8%, 43.2%, respectively; and we found the mortality rates in these groups to be 65.4%, 63.6%, and 52%, respectively. In line with these results, we can say that secondary infections increase mortality. As seen in these rates, we can say that dexamethasone poses the least risk of infection and is associated with lower mortality.

In severe COVID-19 cases, breathing is supported with invasive MV support in cases where adequate oxygenation cannot be provided. A study conducted by Richardson et al. [19] revealed that the mortality of COVID-19 patients, who received IMV, was as high as 88.1%, and that it was significantly higher than those who did not receive MV. In our study, mortality was significantly higher in patients who underwent invasive MV at 84.5% and 86.2% in the steroid and non-steroid groups respectively, compared to the patient group which did not undergo MV. High mortality in MV increases due to secondary infections and complications secondary to MV. For this reason, we believe that strict compliance to infection control measures and applying protective lung ventilation in patients, who will undergo MV, will positively contribute to the reduction of mortality.

ICUs are the departments where hospital infections and resistant infections are observed the most, and the immunosuppressive agents used in these units further increase the risk of secondary infection and cause reproduction of different agents. In their study, Kim et al. [20] found that secondary bacterial pneumonia due to *Acinetobacter baumannii*, *S. aureus* or *Klebsiella pneumoniae* or invasive fungal infection due to invasive pulmonary aspergillosis were more common in the steroid group than in the non-steroid group. In our study, pathogen growths such as *Acinetrobacter*, *S. aureus*, *Klebsiella*, *Pseodomonas*, *Enterobacter* were observed in both groups and there was no statistically significant difference between them.

As regards the meta-analysis of 44 studies by van Paassen et al. [17], it was determined that steroids decreased mortality in the analysis of 22 studies using corticosteroids, steroids had positive effects on the need and duration of MV in 14 studies, and there were more secondary infections and a need for more antibiotics. In our study, the duration of antibiotic treatment was found to be statistically significantly higher in the steroid group. The Langarizadeh et al. [21] study reports that a number of corticosteroids, including methylprednisolone and dexamethasone, demonstrated remarkable efficacy, especially for COVID-19 patients who were mechanically ventilated.

In our study, age and comorbidity rate in the steroid group were found to be statistically significantly lower than in the non-steroid group. Dementia, malignancy, and atrial fibrillation rates were found to be lower in the steroid group than in the non-steroid group. We attribute this to the fact that the steroid group was younger. We believe that the facts that steroids were not used and mostly older patients were admitted in ICU during the first peak and that the curfew for older people, combined with the vaccines being administered after the second peak and especially in the third peak where steroids were most commonly administered, resulted in less ICU visits.

Side effects of steroids can include increased risk of infection, fluid retention, blurred vision, psychological changes, insomnia, and weight gain. In terms of endocrine metabolism, side effects such as hyperglycemia, osteoporosis and osteopenia, dyslipidemia, central obesity, and adrenal suppression may occur [22]. Steroids are the main cause of drug-induced hyperglycemia [23]. Glucocorticoids not only exacerbate hyperglycemia in patients with known DM, but may also cause DM in patients without documented hyperglycemia [24]. In the treatment of hyperglycemia caused by steroids, insulin can be used alongside oral hypoglycemic drugs [25]. In ICUs, insulin is primarily preferred in the treatment of steroid-induced hyperglycemia. In our study, the incidence of DM in our patients was 86 (28%). Therefore, we believe that steroids should be used with caution in these patients in terms of side effects since DM is a common comorbidity.

Guo et al. [26], in their study, revealed that diabetes should be considered as a risk factor for rapid progression and poor prognosis of COVID-19. In our study, the mortality rates in patients with hypertension (HT) and DM who were in the steroid group among the patients who underwent MV were (64.1%) and (72.4%), respectively. In patients without HT and DM, these rates were (49.5%) and (49.6%), respectively, and hence statistically significantly higher. Therefore, we should consider the side effects of steroids and use them more carefully, especially in patients with comorbidities such as DM and HT in COVID-19 patients we use steroids.

We often lose severe COVID-19 patients in intensive care due to complications caused by multiple organ failure which develop secondary to a cytokine storm, or complications which develop due to secondary infections. Taking these two situations under control may be one of the most important steps that will enable us to prevail in this struggle. The fact that steroids are cost-effective and easily available and that we have been using them for many different reasons for a long time has peaked the interest in them in the fight against cytokine storm. We believe using these drugs with the necessary precautions to prevent secondary infections caused by steroids will contribute more to the reduction of mortality. In our study, there was no difference between mortality rates in both groups that took and did not take steroids (Table 4). However, when looking at the ex patients of both groups, we can clearly see that the mortality in the steroid group was 75 (68.2%), while it was 38 (50.7%) in the non-steroid group and we can observe the effect of secondary infections on mortality. In those without reproduction in the same table; mortality was 22 (25.9%) in the steroid group and 37 (49.3%) in the non-steroid group, which indicates an almost 50% less mortality in the steroid group. We believe that this result is associated with steroids extenuating the effect of hyper-inflammatory process which causes multiple organ failure. On the other hand, the increase in secondary infections increases mortality to the same level as the non-steroid group. As a result, we can safely say that while steroids decrease mortality, at the same time they increase mortality due to different reasons.

### Conclusion

It is accepted that cytokine storm, which develops in COVID-19 patients, plays an important role in the damage to the lungs and the development of ARDS. Steroids can be useful in controlling this hyper-inflammatory situation. However, the increase in secondary infections, which is an important side effect of steroids, increases mortality. Steroids more often cause infections of this kind, especially in patients undergoing invasive procedures such as invasive MV and central venous catheter application. Strict adherence to infection control measures at the time of administering steroid treatment to these patients will reduce this risk. In conclusion, while steroids reduce mortality with their positive effects in terms of controlling the hyper-inflammatory picture, they also increase mortality due to increased secondary infections. Finally, we can be more successful with steroids if we can prevent infections.
